# Coding motion direction by action potential patterns

**DOI:** 10.1186/1471-2202-12-S1-P33

**Published:** 2011-07-18

**Authors:** Navid Khosravi, Eric S Fortune, Maurice J Chacron

**Affiliations:** 1 Department of Physiology, McGill University, Montreal, QC, Canada; 2 Department of Psychological and Brain Sciences, Johns Hopkins University, Baltimore, MD, USA; 3 Department of Neuroscience, Johns Hopkins University, Baltimore, MD, USA; 4 Department of Physics, McGill University, Montreal, QC, Canada

## 

Directional selectivity, in which neurons respond more strongly to an object moving in a given direction (‘preferred’) than to the same object moving in the opposite direction (‘null’), is an critical computation achieved in brain circuits as it is a correlate of movement perception [1]. Previous studies have almost exclusively characterized directional selectivity by computing the firing rate, which weights each action potential equally. However, neuronal spike trains frequently contain more or less stereotyped action potential patterns such a bursts (i.e. packets of action potentials followed by quiescence). A growing body of evidence suggests that these patterns, rather than being just a collection of action potentials, can be considered as a unitary event that encodes specific stimulus features (see [2] for review). However, as these studies used stationary stimuli, the putative role of action potential patterns in coding for movement direction remains largely a mystery to this day. To investigate this interesting problem, we performed *in vivo* recordings from direction selective midbrain electrosensory neurons in the weakly electric fish *Apteronotus leptorhynchus* in response to moving objects. We found that most neurons responded to these objects with a combination of bursts and isolated spikes. Segregating the spike train into bursts and isolated spikes revealed that bursts could carry specific information about movement direction. Indeed, in some neurons, we found that bursts were selectively elicited in the preferred direction while isolated spikes were elicited equally in both directions. However, in other neurons, we found that bursts were mostly elicited in one movement direction while isolated spikes were preferentially elicited in the opposite direction. However, when looking at the full spike train, these neurons displayed little or no directional selectivity. In order to explain these surprising results, we built a mathematical model incorporating the previously established mechanisms that generate directional selectivity in TS neurons, namely the generation of a directional bias by in which different time constants of Short Term Depression (STD) across the receptive field and nonlinear integration of these inputs by a subthreshold T-type calcium conductance [3,4]. We found STD time constant ratio values for which our model could reproduce both experimentally observed neuron types. Our results show that action potential patterns can be used to code for movement direction and raise the interesting possibility that bursts and isolated spikes can be used to distinguish movement direction simultaneously in individual neurons. Moreover, our results show that neurons that do not display directional selectivity when their full spike trains are considered can actually display directional selectivity when action potential patterns such as bursts and isolated spikes are considered instead.
